# Complete Genome Sequence of Nervous Necrosis Virus Isolated from Orange-Spotted Grouper (*Epinephelus coioides*) in Taiwan

**DOI:** 10.1128/genomeA.00942-17

**Published:** 2017-09-14

**Authors:** Ming-Hui Chien, Thi Tuyet Minh Vo, Shaw-Yun Wu, Cheng-Hui Lin

**Affiliations:** a Department of Aquaculture, National Taiwan Ocean University, Keelung, Taiwan; b Fisheries Agency, Council of Agriculture, Executive Yuan, Taipei, Taiwan

## Abstract

The genome sequence of nervous necrosis virus strain KS1 isolated from orange-spotted grouper (*Epinephelus coioides*) was cloned and analyzed. The viral genome is composed of two single-stranded positive-sense RNA molecules, RNA1 and RNA2. Phylogenetic analysis shows that the virus strain KS1 belongs to the red-spotted grouper nervous necrosis virus genotype.

## GENOME ANNOUNCEMENT

Viral nervous necrosis (VNN) is one of the most serious diseases in more than 50 species of cultured marine fishes worldwide (1–3). The causative agents of VNN are nervous necrosis viruses (NNVs), belonging to the genus *Betanodavirus* within the family *Nodaviridae* (4). NNV-infected fish show abnormal swimming behavior, such as spiraling and vacuolization (5). NNV has two single-stranded positive-sense RNA molecules, RNA1 and RNA2, which encode RNA-dependent RNA polymerase and capsid protein, respectively. Based on the T4 region of the RNA2 sequence, betanodaviruses are classified into five genotypes: striped jacked NNV (SJNNV), tiger puffer NNV (TPNNV), barfin flounder NNV (BFNNV), red-spotted grouper NNV (RGNNV), and turbot NNV (TNNV) (6–8).

The NNV strain KS1 was isolated from NNV-infected orange-spotted grouper larvae collected from a hatchery farm in Kaohsiung, Taiwan. The complete genome of NNV strain KS1 was amplified by use of a GeneRacer kit (Invitrogen). Then, the PCR products were purified and cloned into pGEM-T easy cloning vectors (Promega). The DNA sequences of all constructs were analyzed by DNA sequencing (Applied Biosystems).

The RNA1 is composed of 3,100 nucleotides and contains an open reading frame (ORF) encoding RNA-dependent RNA polymerase (RdRp). RdRp is composed of 981 amino acids, with a calculated molecular mass of 107.9 kDa. The RNA2 is composed of 1,433 nucleotides and contains an ORF encoding capsid protein. Capsid protein is composed of 338 amino acids, with a calculated molecular mass of 37.1 kDa.

Phylogenetic tree analysis shows that NNV strain KS1 belongs to the RGNNV genotype of betanodavirus. The RNA1 and RNA2 of the NNV strain KS1 had the highest identities, of 98.3% and 99.7%, respectively, with seven-band grouper nervous necrosis virus SGYeosu08 (9). This genome information will facilitate further study of the molecular diagnostics of epizootic and natural host specificity and piscine nodavirus pathogenesis.

### Accession number(s).

The complete genome sequence of the NNV strain KS1 isolated from orange-spotted grouper has been deposited in GenBank under the accession numbers MF144241 (RNA2) and MF144242 (RNA1).
